# *In situ* assessment of statins’ effect on autophagic activity in zebrafish larvae cardiomyocytes

**DOI:** 10.3389/fcvm.2022.921829

**Published:** 2022-11-17

**Authors:** Jie Zhang, Zhi Zuo, Jianxuan Li, Ying Wang, Jia Huang, Lili Xu, Kejia Jin, Hao Lu, Yuxiang Dai

**Affiliations:** ^1^Department of Cardiology, Zhongshan Hospital, Shanghai Institute of Cardiovascular Diseases, Fudan University, Shanghai, China; ^2^Department of Radiology, Shanghai General Hospital, Shanghai Jiao Tong University School of Medicine, Shanghai, China; ^3^Department of Cardiology, The First Affiliated Hospital of Nanjing Medical University, Nanjing, China; ^4^National Clinical Research Center for Interventional Medicine, Shanghai, China; ^5^Fudan Zhangjiang Institute, Shanghai, China

**Keywords:** autophagy, myocardial cell, zebrafish, statins, animal model

## Abstract

Improving the survival rate of cardiomyocytes is the key point to treat most of the heart diseases, and targeting autophagy is a potential advanced therapeutic approach. Monitoring autophagic activity in cardiomyocytes *in situ* will be useful for studying autophagy-related heart disease and screening autophagy-modulating drugs. Zebrafish, *Danio rerio*, has been proven as an animal model for studying heart diseases *in situ*. Taken the advantage of zebrafish, especially the imaging of intact animals, here we generated two stable transgenic zebrafish lines that specifically expressed EGFP-map1lc3b or mRFP-EGFP-map1lc3b in cardiomyocytes under the promoter of myosin light chain 7. We first used a few known autophagy-modulating drugs to confirm their usefulness. By quantifying the density of autophagosomes and autolysosomes, autophagy inducers and inhibitors showed their regulatory functions, which were consistent with previous studies. With the two lines, we then found a significant increase in the density of autophagosomes but not autolysosomes in zebrafish cardiomyocytes at the early developmental stages, indicating the involvement of autophagy in early heart development. To prove their applicability, we also tested five clinical statins by the two lines. And we found that statins did not change the density of autophagosomes but reduced the density of autolysosomes in cardiomyocytes, implying their regulation in autophagic flux. Our study provides novel animal models for monitoring autophagic activity in cardiomyocytes *in situ*, which could be used to study autophagy-related cardiomyopathy and drug screening.

## Introduction

Cardiomyocytes, driving heart contraction, are incapable of cell division in adult mammals (1, 2). Various types of diseases, such as myocardial infarction, hypertension, and hyperlipidemia, result in irreversible loss of cardiomyocytes in acute or chronic ways (3, 4). The subsequent progression stage of cardiomyocyte deficiency is heart failure, which is the leading cause of mortality worldwide and imposes the greatest burden on the healthcare system (5, 6). Due to cardiomyocytes with high demand for energy, many studies reveal that mitochondria and mitochondria-associated ER membranes (MAMs) play important roles in maintaining the metabolism and function of cardiomyocytes, including ATP supply, and the homeostasis of ROS, lipid, and calcium (7–9). And it also has been proven that MAM proteins are involved in the occurrence of heart diseases, thus developing a strategy to eliminate the disordered mitochondria and regulate MAM formation and function might be a promising way to treat those heart diseases (9).

Autophagy, a lysosomal self-degradative process, plays a vital role in maintaining cellular homeostasis (10, 11). It is rapidly activated when cells undergo different stresses, such as starvation, oxidative stress, metabolic stress, and so on (12). In physiological conditions, autophagy maintains cardiac structure and function by degrading misfolded proteins and dysfunctional organelles (13, 14). Autophagy that targets mitochondria, named mitophagy, is critical to eliminate disordered mitochondria and regulate MAMs in cardiomyocytes both at the baseline level and in response to stress (15). In many pathological conditions, autophagy reduces the death of cardiomyocytes by supplying substrates for ATP regeneration in a non-selective or selective bulk degradation pathway (13, 16–18). However, in some other pathological conditions, autophagy in cardiomyocytes is suppressed or activated excessively, and it also contributes to the progression of these heart diseases (19–22). Thus, autophagy plays an essential role in the homeostasis and survival of cardiomyocytes under both basal and stress conditions, which promote the investigators to develop it as a promising therapeutic target (23–25).

Autophagy involves in the formation of double-membrane vesicles called the autophagosome, which sequesters cellular cargo, such as protein aggregates, organelles, ribosomes, and so on. Then, the fusion of the autophagosome with lysosome forms autolysosome, which degrades autophagosomal contents by lysosomal acid proteases (26, 27). The entire process from initiation to the eventual cargo degradation is defined as autophagic flux (28). Microtubule-associated protein 1 light chain 3 (MAP1LC3/LC3) is commonly used as an autophagic marker (29), and its phosphatidylethanolamine (PE) modified counterpart (LC3-PE/LC3-II) specifically localizes to the autophagosomal membranes. LC3-II on the outer autophagosomal membrane is removed in a process called deconjugation, and LC3-II on the inner autophagosomal membrane is degraded by lysosomal enzymes after fusion with lysosome (30). According to such characteristics, LC3-II can be used to indicate the formation and clearance of autophagosomes.

Green fluorescent protein (GFP)-tagged LC3 (GFP-LC3) has been applied to monitor the autophagosomes in living cells and model organisms (31–33). In zebrafish, enhanced GFP (EGFP)-tagged map1lc3b (EGFP-LC3), the zebrafish homolog of human MAP1LC3B, has been reported to study the autophagic function successfully *in situ* (34–36). These studies also point out that the increase in GFP-positive autophagosomes does not necessarily represent the increase in autophagy flux. Interestingly, the tandem monomeric red fluorescent protein (mRFP)-GFP-LC3 can be used to assess the autophagic flux partially, because GFP, not mRFP, is pH sensitive and the fluorescence intensity of GFP dramatically decreases when the fusion of the autophagosome with lysosome happens (30, 37). However, this method for assessing the autophagic flux has not been tested in zebrafish.

Due to the partial similarities with human heart in development, genetics, and physiology, the zebrafish heart is increasingly used as a model of mammalian cardiac studies (38, 39). Myosin light chain 7 (myl7) is expressed specifically in cardiomyocytes and its promoter is wildly used for driving the strong expression of exogenous genes in cardiomyocytes (40, 41). To study the autophagy in cardiomyocytes, we generated two novel stable transgenic zebrafish lines that expressed EGFP-LC3 and mRFP-EGFP-LC3 under the myl7 promoter. With the two transgenic lines, we first showed that the density of autophagosomes and autolysosomes could be faithfully quantified in zebrafish cardiomyocytes, which was proven by treatment with known autophagy-modulating drugs. Second, compared with 2-dpf (days post fertilization) zebrafish larvae, the density of autophagosomes but not autolysosomes were upregulated at 3 dpf, indicating the roles of autophagy in early heart development. Furthermore, statins, 3-hydroxy-3-methylglutaryl-coenzyme A (HMG-CoA) reductase inhibitors, can reduce the synthesis of cholesterol in the liver, and are broadly used in clinical treatment for kinds of heart diseases by reducing the level of LDL cholesterol in plasma (42, 43). However, statins have been reported to be pleiotropic drugs, such as involvement in autophagic regulation in cardiomyocytes (44–46). Here, we tested five statins by the two lines and found that statins did not change the density of autophagosomes, but significantly decreased the density of autolysosomes in cardiomyocytes of zebrafish larvae, suggesting their regulatory roles in autophagic flux. Thus, we described two novel animal models to monitor the autophagic activity in cardiomyocytes *in situ* that can also be used for screening the potential cardiac therapeutic compounds.

## Results

### Strategy of monitoring autophagic activity in zebrafish cardiomyocytes *in situ*

**FIGURE 1:**
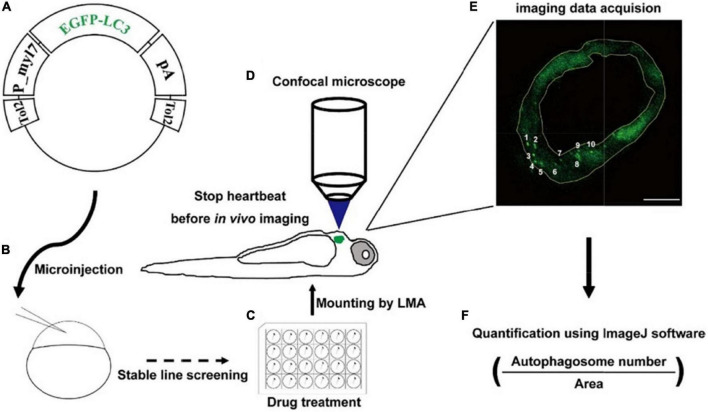
Strategy of monitoring autophagic activity in zebrafish cardiomyocytes *in situ*. **(A)** The structure of transgenic plasmid for labeling autophagosomes. **(B)** Microinjection of the transgenic mixture into one-cell staged embryos for generating stable lines. **(C)** Drug treatment of zebrafish larvae using cell culture plates. **(D)** Transgenic larvae are mounted, stopped heartbeat, and then imaged under a confocal microscope. **(E)** Confocal image shows the EGFP-positive puncta that are characterized as autophagosomes. The area of heart tissue is selected by a yellow polygon using the ImageJ software. Arabic numerals show autophagosomes. Scale bar: 20 μm. **(F)** The density is calculated by dividing the number of autophagosomes by the area of heart tissue.

To examine the autophagic activity in zebrafish cardiomyocytes *in situ*, we first constructed the tol2 transposon-mediated transgenic plasmid, in which the expression of EGFP-LC3 was driven by the promoter of zebrafish myl7 (Figure 1A). Using the plasmid, we generated the stable line Tg(myl7:EGFP-LC3) and found EGFP was only expressed in the heart of zebrafish larvae, which recapitulated the expression of endogenous myl7 transcripts (Figure 1B and Supplementary Figure 1) (40). Using this stable transgenic larvae, we treated them with different drugs (Figure 1C) and then imaged hearts at high resolution *via* confocal microscopy (Figure 1D). In live animals, the imaging of autophagic puncta in cardiomyocytes was largely affected by rhythmic heart beating. Before imaging, we mounted zebrafish larvae with low-melting agarose (LMA) and then used 4% paraformaldehyde (PFA) to fix the larvae or use 2,3-butanedione monoxime (BDM) to stop the heartbeat. Immediately, we imaged the whole heart of zebrafish larvae using Z-stack imaging methods (Figure 1D). At a single optical imaging slice, we could observe distant EGFP-positive puncta that had been characterized as autophagosomes previously in zebrafish, which was compared with broadly EGFP/DsRed expression pattern in Tg(myl7:EGFP) or Tg(myl7:DsRed) transgenic lines (Figure 1E and Supplementary Figure 2) (34). And we also noticed that heart tissue was also labeled by a weak EGFP signal, which could be used for quantifying the area of heart tissue by ImageJ software (Figure 1E). To assess the autophagic activity in a relatively precise way, we chose those slices with at least one EGFP-positive puncta in multiple larvae for the analysis. And then we calculated the density of autophagosomes by dividing the number of autophagosomes by the area of heart tissue (Figure 1F).

### Enhanced green fluorescent protein-labeled autophagosomes in zebrafish cardiomyocytes were regulated by autophagy-modulating drugs

**FIGURE 2:**
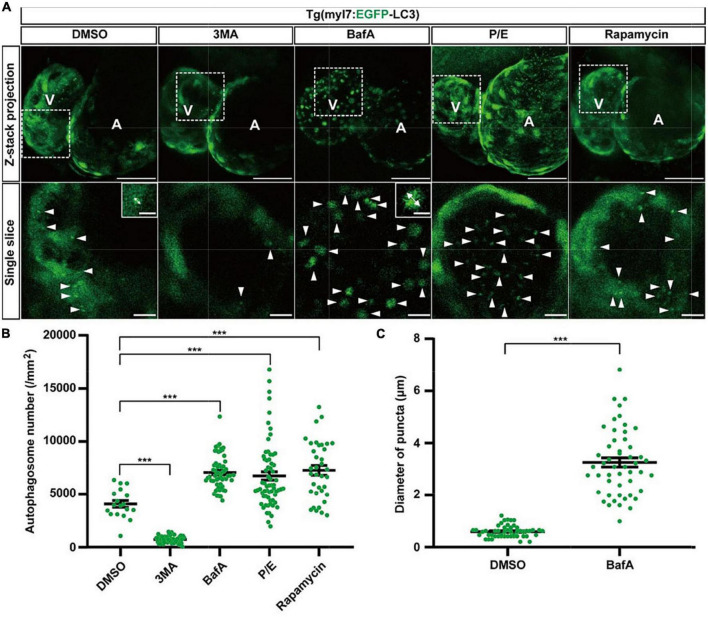
The density of autophagosomes in zebrafish heart is regulated by known autophagy-modulating drugs. **(A)** Representative images of 3-dpf zebrafish heart of Tg(myl7:EGFP-LC3) line when treating with indicated drugs for 24 h. The top row shows the Z-stack maximum projections (scale bar: 50 μm), the bottom row shows the single slice (scale bar: 10 μm), inset in the bottom row shows the single punctum in indicated treated groups (scale bar: 3 μm). Arrowhead represents the autophagosome, double arrow line represents the diameter of the autophagosome. **(B)** Quantification of the density of autophagosomes in 3-dpf zebrafish heart of Tg(myl7:EGFP-LC3) line when treating with indicated drugs for 24 h. The data are shown as mean ± s.e.m for each group. *n* = 18 in control group, *n* = 55 in 3MA group, *n* = 58 in BafA group, *n* = 65 in P/E group, and *n* = 36 in Rapamycin group. **(C)** Quantification of the diameter of puncta in 3-dpf zebrafish heart when treated with BafA compared with the control group. *n* = 50 in the control group, and *n* = 50 in the BafA group. ***p < 0.001. A, atrium; V, ventricle.

To determine the usefulness of the Tg(myl7:EGFP-LC3) line, we first used several small molecules to regulate different stages of autophagic machinery. Using 2-dpf-old transgenic zebrafish larvae, we bathed these molecules, respectively, for 24 h (h). At 3 dpf, EGFP-positive puncta in control zebrafish larvae were mainly located in ventricular cardiomyocytes, and the number of autophagosomes per mm^2^ in cardiomyocytes was 4076.5 ± 323.1 (average ± s.e.m; Figures 2A,B). We first tested 3-methyladenine (3MA), a selective PI3K inhibitor that had been reported to block autophagosome formation (47). Compared with control group, the z-stack projection images showed that 3MA treatment obviously reduced the number of autophagosomes in heart (Figure 2A). The density of autophagosomes in 3MA-treated larvae was significantly decreased by 82% compared to the control (720.6 ± 44.3 per mm^2^, *p*-value = 1.2E-26; Figures 2A,B). Next, we tested other autophagic inhibitor Bafilomycin A1 (BafA), a vacuolar type H + –ATPase (V-ATPase) inhibitor that suppressed autophagosome-lysosome fusion (48), and we found the density of autophagosomes was increased by 73% (7051.8 ± 204.7 per mm^2^, *p*-value = 3.2E-10; Figures 2A,B). And we also noticed that BafA treatment resulted in an obviously larger size of autophagosomes than control (increased by 446%; Figure 2C). We then tested the inhibitors of lysosomal proteases, pepstatin A and E64d (P/E), which prevent the degradation of autophagic cargo inside autolysosomes (27). Similar to BafA, P/E treatment also significantly increased the density of autophagosomes by 65% (6735.5 ± 392.3 per mm^2^, *p*-value = 8.5E-4; Figures 2A,B). Although 3MA, BafA, and P/E could inhibit the autophagic activity in cardiomyocytes of zebrafish larvae, the characteristics of autophagosomes were shown differently including the density and size.

We next test rapamycin, an inhibitor of mTOR, which was reported as an inducer of autophagy (49). Similar to previous studies, rapamycin treatment also increased the density of autophagosomes in cardiomyocytes by 78% compared to the control (7247.4 ± 455.5 per mm^2^, *p*-value = 2.4E-05; Figures 2A,B). To confirm such regulation roles of the autophagy-modulating drugs, we also examined the expression levels of LC3 in WT zebrafish larvae by western blot. We found that 3MA treatment decreased the expression of LC3 significantly, while rapamycin treatment increased, which was consistent with the imaging data by the transgenic line (Supplementary Figure 3). These results show autophagosomes are labeled faithfully by EGFP in the Tg(myl7:EGFP-LC3) line, which could be used for assessing the autophagic activity in cardiomyocytes of zebrafish larvae *in situ*.

### The density of autophagosomes changed in cardiomyocytes during zebrafish’s early developmental stages

**FIGURE 3:**
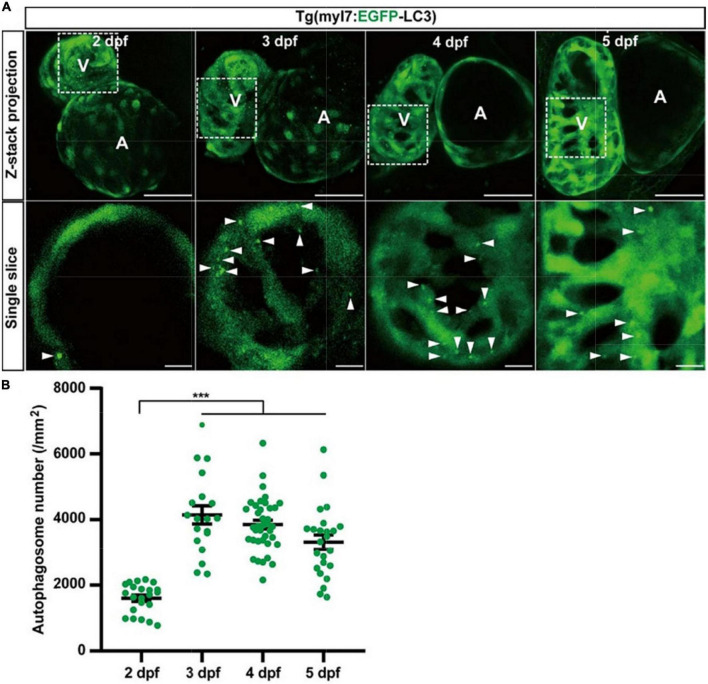
The density of autophagosomes in zebrafish cardiomyocytes is quantified during 2–5 dpf. **(A)** Representative images of zebrafish heart of Tg(myl7:EGFP-LC3) line at indicated developmental stages. Top row shows the Z-stack maximum projections (scale bar: 50 μm), and the bottom row shows the single slice (scale bar: 10 μm). Arrowhead represents the autophagosome. **(B)** Quantification of the density of autophagosomes in zebrafish cardiomyocytes of indicated developmental stages. The data are shown as mean ± s.e.m for each group. *n* = 22 at 2 dpf, *n* = 19 at 3 dpf, *n* = 38 at 4 dpf, and *n* = 24 at 5 dpf. ***p < 0.001. A, atrium; V, ventricle.

To understand autophagy in zebrafish’s early heart development, we imaged the heart of Tg(myl7:EGFP-LC3) zebrafish larvae at different developmental stages. EGFP expression driven by myl7 promoter could be detected as early as 16 h post-fertilization (hpf) (40). However, the EGFP-LC3 signal was too weak to detect before 1 dpf in Tg(myl7:EGFP-LC3) zebrafish larvae. At 2 dpf, the EGFP-LC3 signal could be detected quite easier, and we found the density of autophagosomes was 1601.6 ± 95.7 per mm^2^ (Figure 3). At 3 dpf, the density of autophagosomes was significantly increased by 172% (4143.7 ± 279.6, *p*-value = 7.5E-14; Figure 3) compared to that at 2 dpf. After 3 dpf, the density of autophagosomes is relatively stable (3849.3 ± 132.7 per mm^2^ at 4 dpf; 3314.3 ± 220.6 per mm^2^ at 5 dpf; Figure 3). These data show that autophagic activity changes during zebrafish early heart development, implying the autophagic role in heart development.

### Autophagic flux in zebrafish cardiomyocytes was regulated by autophagy-modulating drugs

**FIGURE 4:**
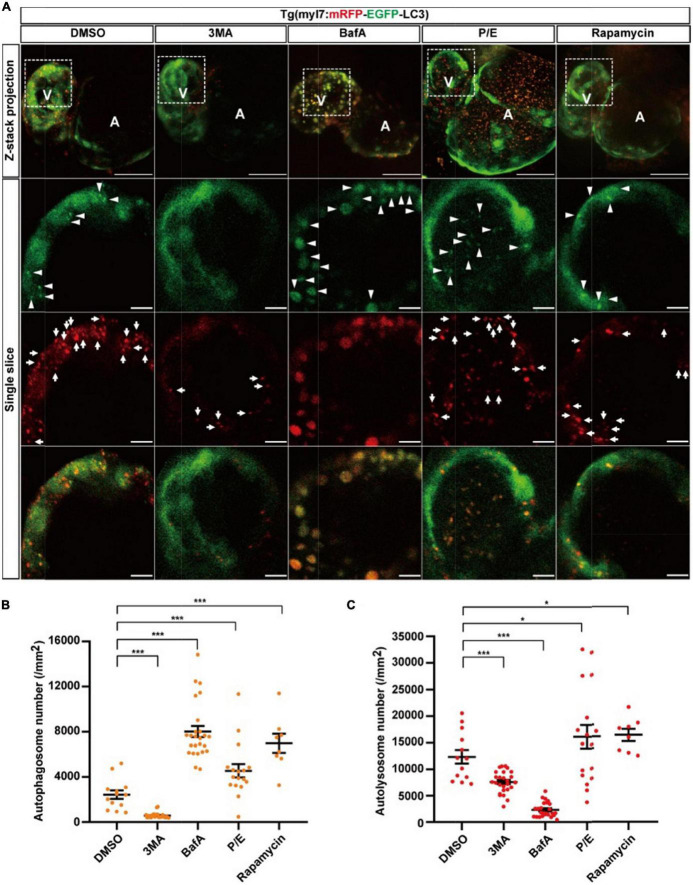
The densities of autophagosomes and autolysosomes in zebrafish cardiomyocytes are regulated by known autophagy-modulating drugs. **(A)** Representative images of 3-dpf zebrafish heart of Tg(myl7:mRFP-EGFP-LC3) line when treated with indicated drugs for 24 h. The top row shows the Z-stack maximum projections with merged EGFP and mRFP signals (scale bar: 50 μm), the second row shows the single slice with EGFP signal, the third row shows the single slice with mRFP signal, and the bottom row shows the single slice with merged EGFP and mRFP signals (scale bar: 10 μm). Arrowhead represents autophagosome, the arrow represents autolysosome. **(B)** Quantification of the density of autophagosomes in 3-dpf zebrafish heart of Tg(myl7:mRFP-EGFP-LC3) line when treated with indicated drugs for 24 h. **(C)** Quantification of the density of autolysosomes in 3-dpf zebrafish heart of Tg(myl7:mRFP-EGFP-LC3) line when treated with indicated drugs for 24 h. The data are shown as mean ± s.e.m for each group. *n* = 13 in control group, *n* = 26 in 3MA group, *n* = 25 in BafA group, *n* = 17 in P/E group, and *n* = 8 in Rapamycin group. *p < 0.05, ***p < 0.001. A, atrium; V, ventricle.

To assess the autophagic flux of cardiomyocytes in intact animals *in situ* instead of lysosome staining by LysoTraker Red in fixed samples (36), we generated another transgenic line Tg(myl7:mRFP-EGFP-LC3), in which EGFP and mRFP were specifically expressed in the heart of zebrafish larvae (Supplementary Figure 1). Similar to Tg(myl7:EGFP-LC3), we used autophagy-modulating drugs to demonstrate their applicability. In the control group, we quantified the puncta with both EGFP and mRFP positive signals representing autophagosomes and found the density of colocalized puncta was 2440.0 ± 376.1 per mm^2^ (Figures 4A,B). The density of puncta with only mRFP positive signal representing autolysosomes was 12344.8 ± 1281.1 per mm^2^ (Figure 4C). Compared with the control, 3MA treatment decreased the density of autophagosomes by 76% (577.8 ± 48.2 per mm^2^, *p*-value = 2.2E-8; Figure 4B), and it also decreased the density of autolysosomes by 39% (7571.5 ± 402.1 per mm^2^, *p*-value = 3.3E-5; Figure 4C). BafA treatment increased the density of autophagosomes by 229% (8022.4 ± 504.4 per mm^2^, *p*-value = 4.8E-9; Figure 4B), but decreased the density of autolysosomes by 81% (2330.2 ± 278.9 per mm^2^, *p*-value = 2.4E-12; Figure 4C). Different from the BafA treatment, the P/E treatment increased the density of autophagosomes by 87% (4555.6 ± 587.2 per mm^2^, *p*-value = 4.3E-3; Figure 4B), and the density of autolysosomes was also increased by 37% (16904.4 ± 2114.5 per mm^2^, *p*-value = 4.9E-2; Figure 4C). Similar to Tg(myl7:EGFP-LC3) line, the BafA1 treatment also resulted in a large size of the puncta than the control (Supplementary Figure 4). Using rapamycin to induce the autophagy increased the density of autophagosomes by 186% (6986.7 ± 837.8 per mm^2^, *p*-value = 9.9E-6; Figure 4B) and autolysosomes by 32% (16347.9 ± 1130.2 per mm^2^, *p*-value = 2.3E-2; Figure 4C). According to the function of BafA and P/E, which were reported previously (27, 48), our data indicate that BafA inhibits the autophagic flux by blocking the fusion of autophagosomes and lysosomes, and P/E inhibits the autophagic flux by preventing the degradation of autophagic cargo inside autolysosomes. These data showed this transgenic line could be applied for assessing the autophagic flux by quantification the density of autophagosomes and autolysosomes simultaneously.

### The density of autolysosomes did not change in cardiomyocytes during zebrafish’s early developmental stages

**FIGURE 5:**
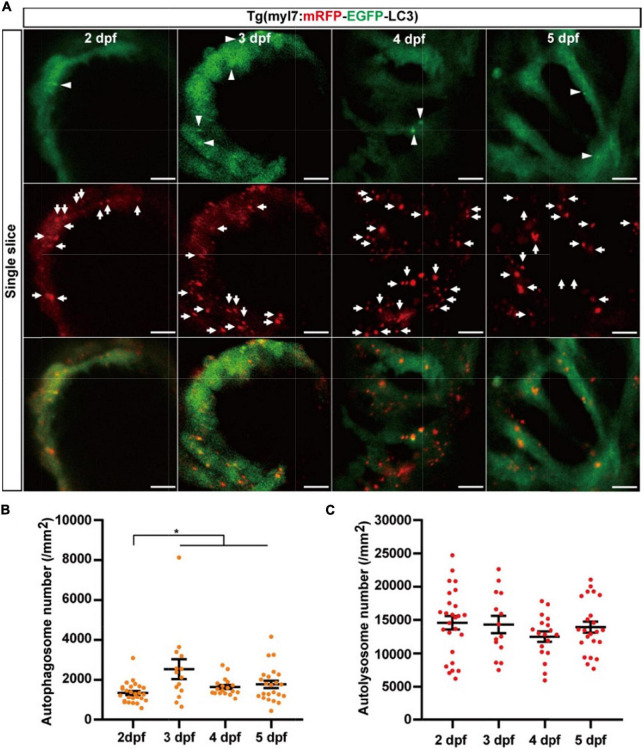
The densities of autophagosomes and autolysosomes in zebrafish hearts are quantified simultaneously during 2–5 dpf. **(A)** Representative images of 3-dpf zebrafish heart of Tg(myl7:mRFP-EGFP-LC3) line at indicated developmental stages. The top row shows the single slice with an EGFP signal, the second row shows the single slice with an mRFP signal, and the bottom row shows the single slice with merged EGFP and mRFP signals (scale bar: 10 μm). Arrowhead represents autophagosomes, the arrow represents autolysosomes. **(B)** Quantification of the density of autophagosomes in zebrafish heart of Tg(myl7:mRFP-EGFP-LC3) line at indicated developmental stages. **(C)** Quantification of the density of autolysosomes in zebrafish heart of Tg(myl7:mRFP-EGFP-LC3) line at indicated developmental stages. The data are shown as mean ± s.e.m for each group. *n* = 26 at 2 dpf, *n* = 14 at 3 dpf, *n* = 18 at 4 dpf, and *n* = 23 at 5 dpf. *p < 0.05. A, atrium; V, ventricle.

We next used Tg(myl7:mRFP-EGFP-LC3) to quantify autophagic flux at zebrafish developmental stages. At 2 dpf, the density of autophagosomes is 1351.9 ± 101.0 per mm^2^), and the density of autolysosomes was 14586.9 ± 999.0 per mm^2^ (Figure 5). Similar to the Tg(myl7:EGFP-LC3) line, the density of autophagosomes was also increased at 3 dpf (2541.2 ± 495.9 per mm^2^, *p* = 1.9E-3; Figure 5B) compared with that at 2 dpf. However, the density of autolysosomes did not change significantly at 3 dpf (14328.1 ± 1297.2 per mm^2^; Figure 5C). After 3 dpf, the density of autophagosomes and autolysosomes were both relatively stable (autophagosome: 1646.8 ± 103.6 per mm^2^ at 4 dpf; 1779.7 ± 184.0 per mm^2^ at 5 dpf; autolysosome: 12504.7 ± 777.2 per mm^2^ at 4 dpf; 13928.1 ± 831.8 per mm^2^ at 5 dpf; Figures 5B,C). Combing the data obtained using Tg(myl7:EGFP-LC3) line, these data reveal that autophagosome but not autophagic flux is increased in zebrafish cardiomyocytes at the early developmental stages.

### Statins regulate autophagic flux in zebrafish cardiomyocytes

**FIGURE 6:**
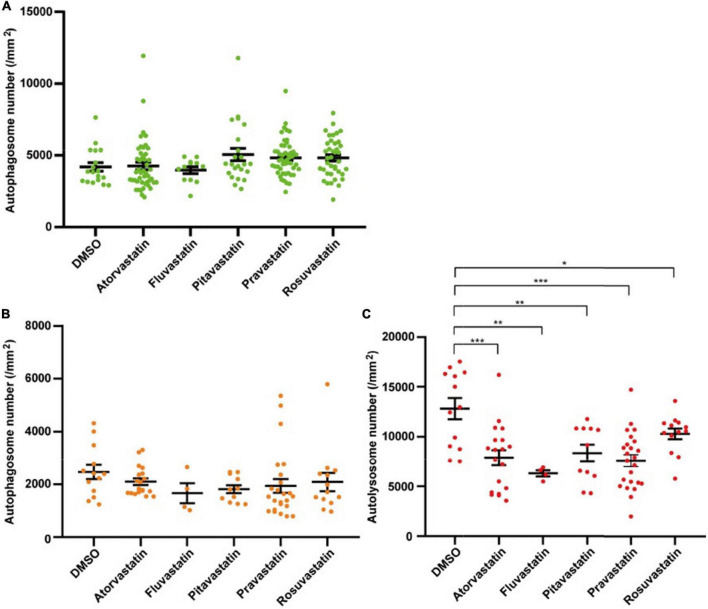
The densities of autophagosomes and autolysosomes in zebrafish cardiomyocytes are quantified when treated with statins. **(A)** Quantification of the density of autophagosomes in 3-dpf zebrafish heart of Tg(myl7:EGFP-LC3) line when treated with indicated statins. **(B)** Quantification of the densities of autophagosomes in 3-dpf zebrafish heart of Tg(myl7:mRFP-EGFP-LC3) line when treated with indicated statins. **(C)** Quantification of the densities of autolysosomes in 3-dpf zebrafish heart of Tg(myl7:mRFP-EGFP-LC3) line when treated with indicated statins. The data are shown as mean ± s.e.m for each group. *n* = 13 in the control group, *n* = 19 in the atorvastatin group, *n* = 4 in the fluvastatin group, *n* = 11 in pitavastatin, *n* = 24 in the pravastatin group, and *n* = 13 in the rosuvastatin group. *p < 0.05, **p < 0.01, ***p < 0.001.

Statins are broadly applied in clinical treatment for different heart diseases (43, 45). To test whether the two lines can be used for studying the autophagy-related drugs in cardiomyocytes, we tested five statins, including atorvastatin, fluvastatin, pitavastatin, pravastatin, and rosuvastatin. We first tested five statins by Tg(myl7:EGFP-LC3) zebrafish line and found that these five statins did not change the density of autophagosomes (4191.1 ± 299.0 per mm^2^ in the control group, 4256.8 ± 358.4 per mm^2^ in the atorvastatin group, 3964.3 ± 238.7 per mm^2^ in the fluvastatin group, 5050.2 ± 436.6 per mm^2^ in pitavastatin, 4823.1 ± 180.0 per mm^2^ in the pravastatin group, and 4822.9 ± 218.9 per mm^2^ in the rosuvastatin group; Figure 6A). Next, we test them by Tg(myl7:mRFP-EGFP-LC3) zebrafish line. Similar to Tg(myl7:EGFP-LC3) line, we also found statins did not change the density of autophagosomes (2470.9 ± 269.1 per mm^2^ in the control group, 2102.3 ± 124.7 per mm^2^ in the atorvastatin group, 1667.8 ± 373.7 per mm^2^ in the fluvastatin group, 1820.2 ± 148.2 per mm^2^ in pitavastatin, 1941.2 ± 258.5 per mm^2^ in the pravastatin group, and 2090.3 ± 345.0 per mm^2^ in the rosuvastatin group; Figure 6B). Interestingly, the density of autolysosomes in cardiomyocytes was decreased when treated with statins (12812.1 ± 1057.7 per mm^2^ in the control group; 7886.0 ± 761.3 per mm^2^ in the atorvastatin group, *p* = 5.3E-4; 6308.1 ± 308.2 per mm^2^ in the fluvastatin group, *p* = 4.6E-3; 8355.8 ± 855.6 per mm^2^ in pitavastatin, *p* = 4.2E-3; 7588.1 ± 582.1 per mm^2^ in the pravastatin group, *p* = 3.7E-5; and 10285.2 ± 542.2 per mm^2^ in the rosuvastatin group, *p* = 4.4E-2; Figure 6C). These results suggest that statins might play roles in the regulation of autophagic flux in cardiomyocytes.

## Discussion

Many studies demonstrate that autophagy is involved in different heart diseases, and targeting autophagic processes has emerged as an effective therapeutic strategy (23–25). Due to the partial similarity especially in the conserved molecular and cellular pathways, the zebrafish heart is increasingly used as a model for studying the function and disease of human hearts. Besides, zebrafish have many advantages among vertebrate models, such as transparent larvae for *in situ* observation, ease of genetic manipulation, and large amount of embryos with low cost for drug screening. Furthermore, it has also been demonstrated that autophagy in cardiomyocytes plays essential role in heart development and regeneration in zebrafish models (50, 51). For these reasons, the developing zebrafish transgenic lines in this study will offer researchers novel models for easily assessing autophagic activities in cardiomyocytes *in situ*.

It has been proved that EGFP-LC3 can be used for monitoring the autolysosomes when it is overexpressed in zebrafish ubiquitously, or specifically in hepatocytes or neurons (35, 36). For studying cardiomyocyte-specific autophagy, we generated the first transgenic zebrafish model that EGFP-LC3 specifically overexpressed in cardiomyocytes. To maximize the benefits of *in situ* autophagy-related research, we also generated another zebrafish model that mRFP-EGFP-LC3 specifically overexpressed in cardiomyocytes. After testing the known autophagy-modulating drugs, we validated the usefulness of the two lines for labeling the autophagosomes and autolysosomes. In the two lines, we found the change in autophagic activity in cardiomyocytes happened at the developmental stages, which was consistent with the notion that autophagy played a critical role in heart development (50). Especially, autophagic activity in zebrafish cardiomyocytes showed a significant change within 2 dpf and 3 dpf (Figures 3, 5). During this stage, it is reported that internal cardiac structure changes, such as the zebrafish heart valve and conduction system (38, 52, 53). During 55–72 hpf, the retrograde blood flow has ceased gradually, and pacemaker activity becomes restricted to a small area on the dorsal right quadrant of the sinoatrial ring (52, 53), which implies autophagic activity in cardiomyocytes might be regulated by hemodynamics and/or cardiac conduction system. And we also found the number of EGFP labeled autophagosomes in Tg(myl7:EGFP-LC3) line was much higher than that in Tg(myl7:mRFP-EGFP-LC3) line, but the changes in autophagic activities showed more sensitive in Tg(myl7:mRFP-EGFP-LC3) than that in Tg(myl7:EGFP-LC3) line when treating with autophagy-modulating drugs. Recently, Mareninova et al. reported that GFP-LC3 overexpression could affect autophagy itself (54). Although we did not observe any abnormal phenotype of the heart in at least five generations of the two transgenic zebrafish, it still reminds us to develop better *in situ* autophagic models in the future.

The rate of autophagic degradation is defined as autophagic flux, which includes the entire process of autophagy. Usually, assessing autophagic flux requires the combination of multiple techniques, but they cannot be achieved in intact animals *in situ* (27, 55). By examining the changes of both autophagosomes and autolysosomes simultaneously, we could partially assess the autophagic flux. After testing different autophagy-modulating drugs by Tg(myl7:mRFP-EGFP-LC3) line, we found that 3MA treatment reduced the density of both autophagosomes and autolysosomes, which was consistent with its inhibitory role of autophagosome formation. BafA treatment resulted in the increase of autophagosomes but a decrease in autolysosomes, which was also consistent with its inhibitory role of autophagosome-lysosome fusion. However, the increase of both autophagosomes and autolysosomes has resulted from increased autophagic activity or decreased degradation in autolysosomes. In the present study, treating rapamycin showed a similar increase to that of P/E. To distinguish such situations, different known autophagy inhibitors can be used in combination (27, 36). Besides, the rate of autophagosome/autolysosome accumulation also can be used for the evaluation (36). Due to the autophagic interference when blocking heartbeat (13), the methods for quantifying the rate of autophagosome/autolysosome accumulation in cardiomyocytes still need to be developed. In addition, we noticed that the size of autophagosomes is enlarged when treated with BafA, which could be a novel parameter for the assessment of autophagic flux.

One of the advantages of the zebrafish models is the application of *in situ* drug development. Statins can effectively lower the cholesterol level in the blood by inhibiting cholesterol synthesis, and then achieve therapeutic effects for heart diseases (45). They also have been reported to be involved in autophagic regulation, but their mechanism in autophagic regulation is still unclear (46). In the present study, we tested statins initially by the generated transgenic lines at the highest concentration with no side effects in zebrafish larvae. Although it might not be the optimal concentration to regulate the autophagy in the cardiomyocytes of zebrafish larvae, we still found their regulatory roles in our transgenic lines. Different from the known autophagy-modulating drugs, statins only significantly decreased the density of autolysosomes but not autophagosomes. Based on the data, we speculate that statins might promote autophagic flux in cardiomyocytes by regulating the degradation process in autolysosomes, suggesting the feasibility of autophagic drug development in cardiomyocytes by zebrafish transgenic models.

In this study, we developed two transgenic lines that could be used to determine the density of autophagosomes and autolysosomes in zebrafish cardiomyocytes. With these lines, we found the changes in autophagic activity that happened in cardiomyocytes at early zebrafish developmental stages. In the applicability test, we found that statins could play roles in the regulation of autophagic flux. Taken together, we believe that these zebrafish lines would be useful for autophagy-related studies for cardiomyopathy. With the help of our *in situ* vertebrate models, we hope it would alleviate the stress of the healthcare system by accelerating the development of autophagy-related drugs for cardiomyopathy.

## Materials and methods

### Zebrafish care and maintenance

Zebrafish were maintained at 28.5^°^C on a 14 h/10 h light/dark cycle with automatic fish housing system (Haishen, China). Embryos and larvae were raised in 10% Hank’s solution that consisted of (in mM) 140 NaCl, 5.4 KCl, 0.25 Na_2_HPO_4_, 0.44 KH_2_PO_4_, 1.3 CaCl_2_, 1.0 MgSO_4_, and 4.2 NaHCO_3_ (pH 7.2). To prevent pigment formation for imaging experiments, the embryos were treated with 0.003% 1-phenyl-2-thiourea (PTU, Sigma) from 24 h post-fertilization. AB wild-type was used for generating novel transgenic lines. Tg(myl7:EGFP) and Tg(myl7:DsRed) transgenic lines were used as the negative line for identifying the autophagic puncta. Zebrafish handling procedures were approved by the Animal Research Committee of Zhongshan Hospital, Fudan University.

### Transgenic plasmid construction

The transgenic plasmids were constructed by Hieff Clone^®^ Plus Multi One Step Cloning Kit (YEASEN, China). The 269-bp promoter of myl7 was amplified from zebrafish genomic DNA. The coding sequence of zebrafish map1lc3b was amplified from the cDNA of 3-dpf zebrafish larvae. EGFP or mRFP sequences were purchased from the Miaolingbio Company (China). Different fragments were ligated in the tol2mini transgenic vector (56).

### Generation of Tg(myl7:EGFP-LC3) and Tg(myl7:mRFP-EGFP-LC3) transgenic zebrafish

One-cell staged AB zebrafish embryos were collected by natural mating and were injected with 25 pg of myl7-EGFP-LC3 along with 25 pg tol2 transposase mRNA. The founders of Tg(myl7:EGFP-LC3) were screened by EGFP expression of their F1 progenies, and F1 larvae were then confirmed by genotyping. Similarly, stable lines of Tg(myl7:mRFP-EGFP-LC3) were screened out. In total, we identified six different alleles of the two transgenic lines. The alleles with strong expression were selected by MVX10 stereoscopic fluorescence microscope (Olympus, Japan) for further studies. All stable lines have been outcrossed for at least five generations with no abnormalities at developmental stages or adulthood (data not shown).

### Drug treatment of zebrafish larvae

Autophagy-modulating drugs and statins were all purchased from Selleck Company (China). Dechorionated 2-dpf transgenic embryos were incubated with different drugs for 24 h. We tested different concentrations of each drug, and the groups with all larvae developed normally were considered as having no side effects. And we chose the highest concentration group with no side effects of each drug for further autophagy imaging experiments (Supplementary Table 1). The larvae treated with 10 mM 3-methyladenine (3MA, S2767), 200 nM bafilomycin A1 (BafA, S1413), P/E: 10 μg/mL pepstatin A (S7381) and 5 μg/mL E64d (Aloxistatin, S7393), 1 μM rapamycin (S1039), 10 μg/mL Atorvastatin (S5715), 8 μg/mL Fluvastatin (S1909), 4 μg/mL Pitavastatin (S1759), 9 μg/mL Pravastatin (S5713), or 10 μg/mL Rosuvastatin (S2169), respectively, were used for the autophagy imaging experiments. All drugs were diluted in 10% Hank’s solution. The final concentration of DMSO in all groups including the control group was 0.1% (v/v). Six-well plates were used for the drug treatment. Not more than 10 embryos were treated in each well containing 4 mL of treatment solution.

### Confocal imaging and quantification of puncta

For *in situ* confocal imaging, larvae were immobilized in 1.2% low melting-point agarose into 35-mm glass-bottom culture dishes without anesthetics. The heartbeat of mounted larvae was stopped by adding 4% paraformaldehyde (PFA) or 20 mM 2,3-butanedione 2-monoxime (BDM) in culture dishes. An imaging experiment of each larva would be completed within 10 min after the heartbeat stopped. Z-stack imaging of the entire heart of transgenic larvae with the ventral view was acquired using a 488/594 nm excitation lasers of Fluoview 3000 confocal microscope (Olympus, Japan). Imaging experiments were performed under a 20X dry objective with zoom in x3.0. The spatial resolution of all images was 1,024 × 1,024 pixels with a z-step size of 4 μm.

Fluorescent points (EGFP + or mRFP +) which were distinguishable from the background were considered puncta. The area of heart tissue was quantified based on the weak EGFP signals by the manual polygon selection using ImageJ software. Numbers of puncta and areas of heart tissue were quantified from each z-stack slice. The slices without EGFP + puncta were excluded from the quantification. The data point from each zebrafish larva varied from 0 to 5. EGFP + or EGFP + /mRFP + points were considered autophagosomes, and EGFP-/mRFP + points were considered autolysosomes.

### Western blot

Wild-type zebrafish larvae treated with autophagic regulation drugs (3MA and rapamycin) were used for total protein extraction. About 40 3-dpf wild-type zebrafish larvae were lysed by Radio Immunoprecipitation Assay Lysis buffer (RIPA, Beyotime, China) for total protein extraction. The protein samples were separated by sodium dodecyl sulfate–polyacrylamide gel electrophoresis and electro-transferred on polyvinylidene fluoride membranes (Millipore, Schwalbach, Germany), then were blocked with 5% skim milk. Subsequently, the membranes were incubated with primary antibody (BOSTER, China) for LC3B/MAP1LC3B (BM4827, 1:500) or GAPDH (BM1623, 1:10,000) at 4°C overnight. Then the membranes were put into the secondary antibody (BOSTER, China) HRP Conjugated AffiniPure Goat Anti-Mouse IgG (H + L) (BA1050, 1:5000) or Anti-Rabbit (BA1054, 1:5,000) for 1 h at room temperature. After washing, the proteins were visualized with a BeyoECL plus kit (Beyotime, China).

### Statistical analysis

Statistical analysis was performed using Microsoft Excel or GraphPad Prism version 8.0.1. Student’s *t*-tests were used to compare two groups. One-way ANOVA analyses were used for comparisons among multiple groups followed by Tukey’s multiple comparisons tests. All results are represented as mean ± SEM. GraphPad Prism software was used to generate the statistical graphs.

## Data availability statement

The original contributions presented in this study are included in the article/Supplementary material, further inquiries can be directed to the corresponding author/s.

## Ethics statement

This animal study was reviewed and approved by the Animal Ethics Committee, Zhongshan Hospital, Fudan University.

## Author contributions

YD and HL: conceptualization, methodology, supervision, project administration, and funding acquisition. JZ and ZZ: formal analysis and investigation. JZ, ZZ, and JH: data curation. JZ, YW, and JH: writing—original draft preparation. LX and KJ: writing—review and editing. All authors have read and agreed to the published version of the manuscript.
