# Supplementary material for: In situ assessment of statins’ effect on autophagic activity in zebrafish larvae cardiomyocytes
Source: Front Cardiovasc Med. 2022 Nov 17;9:921829. doi: 10.3389/fcvm.2022.921829 (PMC9712203; doi:10.3389/fcvm.2022.921829)
Supplement: Supplementary file 1 [file Data_Sheet_1.docx]

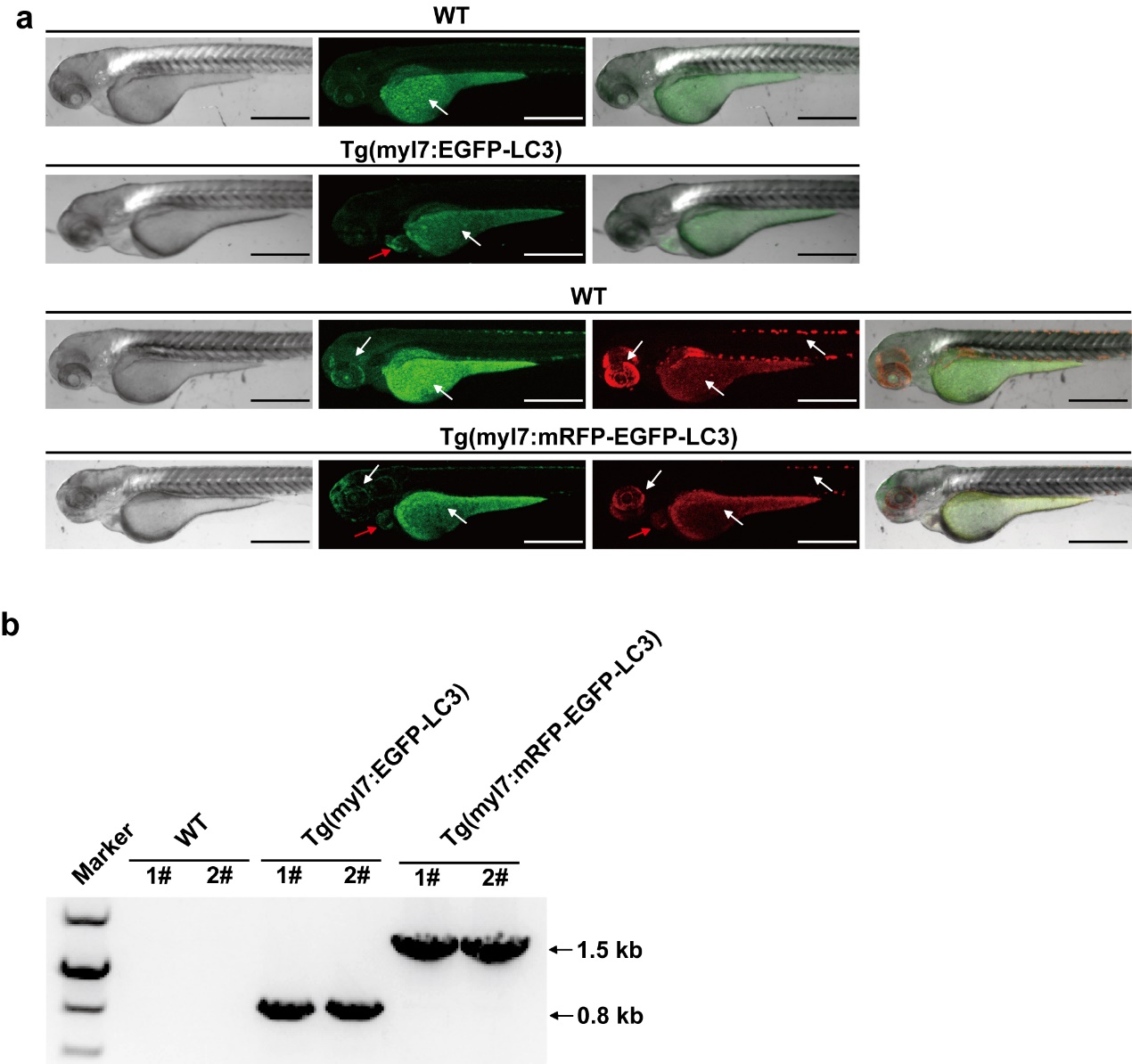


**Figure S1.** Screening for Tg(myl7:EGFP-LC3) and Tg(myl7:mRFP-EGFP-LC3) stable lines. (a) The phenotype of 3-dpf larva of Tg(myl7:EGFP-LC3) and Tg(myl7:mRFP-EGFP-LC3) lines compared with wild-type larva at same imaging conditions respectively under the confocal microscope. Note that the specific fluorescent signals are shown by the red arrows, and autofluorescence of the zebrafish skin, yolk and eye are shown by white arrows. Scale bar: 500 µm. (b) Genotyping for Tg(myl7:EGFP-LC3) and Tg(myl7:mRFP-EGFP-LC3) lines.


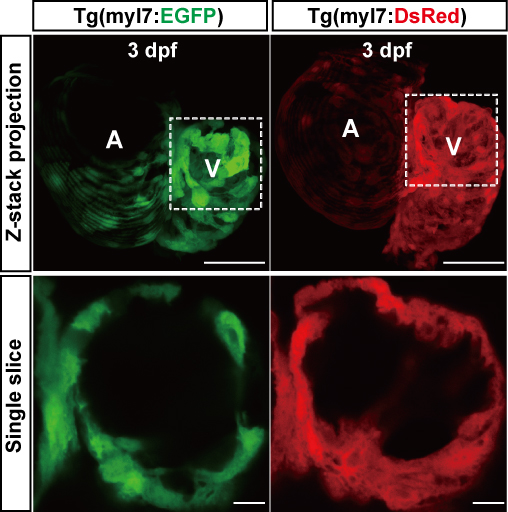


**Figure S2**. Representative images of zebrafish heart of Tg(myl7:EGFP) and Tg(myl7:DsRed) lines at 3 dpf. Top row shows the Z-stack maximum projections (scale bar: 50 µm), bottom row shows the single slice (scale bar: 10 µm).


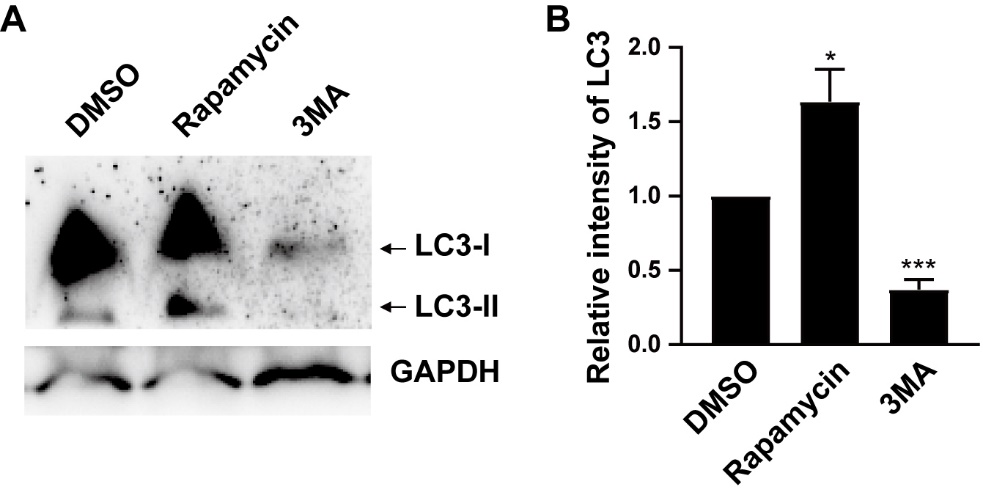


**Figure S3**. Western blot of LC3 of WT zebrafish larvae (A) and summary data of three repeats (B), which treated with DMSO (control), Rapamycin and 3MA.


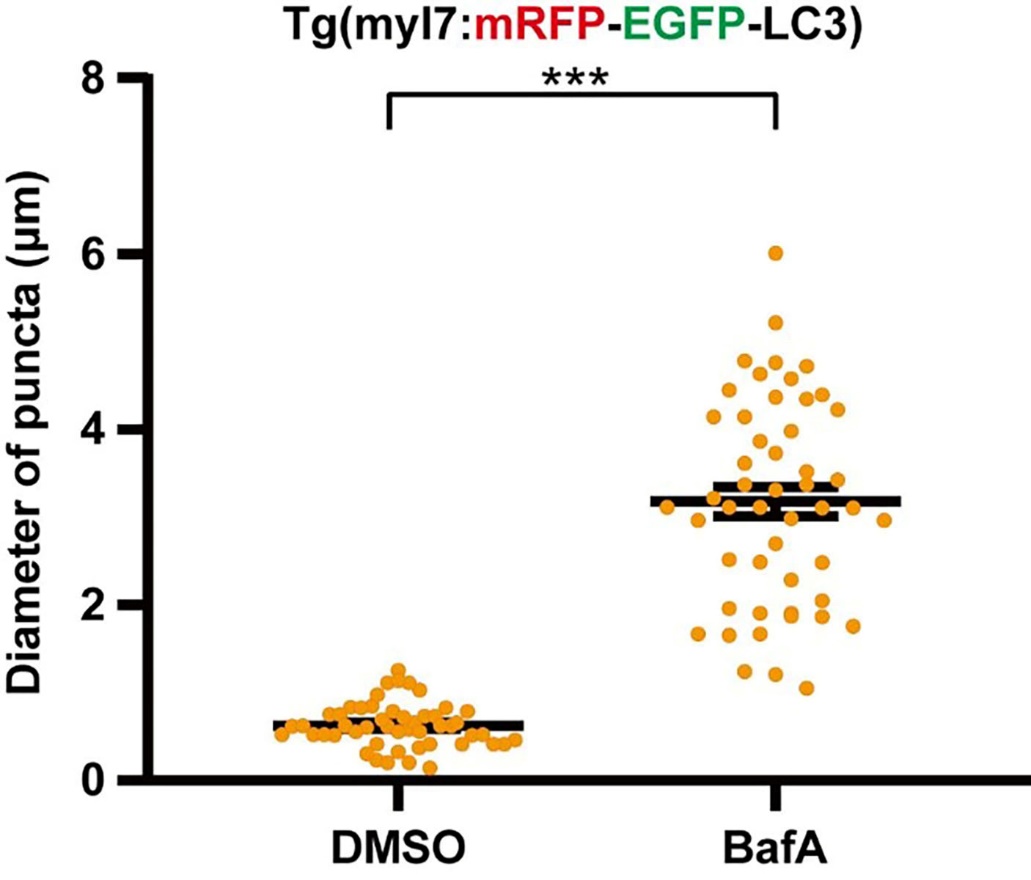


**Figure S4.** Quantification of the diameter of puncta in 3-dpf zebrafish heart in Tg(myl7:mRFP-EGFP-LC3) line when treating with BafA compared with control (DMSO) group. n = 50 in control group, and n = 50 in BafA group. ***: p < 0.001.

**Table S1.** Normal development rate when treated with different concentration of each drug.

| **Drug** | **Concentration** | **The rate of normal development** |
| --- | --- | --- |
| 3MA | 6 mM | 100% (10/10) |
|  | 8 mM | 100% (10/10) |
|  | 10 mM | 100% (10/10) |
|  | 12 mM | 90% (9/10) |
|  | 14 mM | 80% (8/10) |
| BafA | 50 nM | 100% (10/10) |
|  | 100 nM | 100% (10/10) |
|  | 150 nM | 100% (10/10) |
|  | 200 nM | 100% (10/10) |
|  | 250 nM | 70% (7/10) |
| P/E | 1.25 μg/mL, 0.625 μg/mL | 100% (10/10) |
|  | 2.5 μg/mL, 1.25 μg/mL | 100% (10/10) |
|  | 5 μg/mL, 2.5 μg/mL | 100% (10/10) |
|  | 10 μg/mL, 5 μg/mL | 100% (10/10) |
|  | 20 μg/mL, 10 μg/mL | 90% (9/10) |
| Rapamycin | 0.4 μM | 100% (10/10) |
|  | 0.6 μM | 100% (10/10) |
|  | 0.8 μM | 100% (10/10) |
|  | 1 μM | 100% (10/10) |
|  | 1.2 μM | 90% (9/10) |
| Atorvastatin | 2.5 μg/mL | 100% (10/10) |
|  | 5.0 ug/mL | 100% (10/10) |
|  | 10 μg/mL | 100% (10/10) |
|  | 20 μg/mL | 60% (6/10) |
|  | 40 μg/mL | 20% (2/10) |
| Fluvastatin | 2 μg/mL | 100% (10/10) |
|  | 4 μg/mL | 100% (10/10) |
|  | 8 μg/mL | 100% (10/10) |
|  | 16 μg/mL | 90% (9/10) |
|  | 32 μg/mL | 60% (6/10) |
| Pitavastatin | 1 μg/mL | 100% (10/10) |
|  | 2 μg/mL | 100% (10/10) |
|  | 4 μg/mL | 100% (10/10) |
|  | 8 μg/mL | 70% (7/10) |
|  | 16 μg/mL | 10% (1/10) |
| Pravastatin | 1 μg/mL | 100% (10/10) |
|  | 2 μg/mL | 100% (10/10) |
|  | 4.5 μg/mL | 100% (10/10) |
|  | 9 μg/mL | 100% (10/10) |
|  | 18 μg/mL | 90% (9/10) |
| Rosuvastatin | 2.5 μg/mL | 100% (10/10) |
|  | 5 μg/mL | 100% (10/10) |
|  | 10 μg/mL | 100% (10/10) |
|  | 20 μg/mL | 70% (7/10) |
|  | 40 μg/mL | 60% (6/10) |
